# Metamorphosis in the Cirripede Crustacean *Balanus amphitrite*

**DOI:** 10.1371/journal.pone.0037408

**Published:** 2012-05-30

**Authors:** Diego Maruzzo, Nick Aldred, Anthony S. Clare, Jens T. Høeg

**Affiliations:** 1 Department of Biology, University of Padova, Padova, Italy; 2 School of Marine Science & Technology, Newcastle University, Newcastle upon Tyne, United Kingdom; 3 Marine Biology Section, Department of Biology, University of Copenhagen, Copenhagen, Denmark; University of Gothenburg, Sweden

## Abstract

Stalked and acorn barnacles (Cirripedia Thoracica) have a complex life cycle that includes a free-swimming nauplius larva, a cypris larva and a permanently attached sessile juvenile and adult barnacle. The barnacle cyprid is among the most highly specialized of marine invertebrate larvae and its settlement biology has been intensively studied. By contrast, surprisingly few papers have dealt with the critical series of metamorphic events from cementation of the cyprid to the substratum until the appearance of a suspension feeding juvenile. This metamorphosis is both ontogenetically complex and critical to the survival of the barnacle. Here we use video microscopy to present a timeline and description of morphological events from settled cyprid to juvenile barnacle in the model species *Balanus amphitrite*, representing an important step towards both a broader understanding of the settlement ecology of this species and a platform for studying the factors that control its metamorphosis. Metamorphosis in *B. amphitrite* involves a complex sequence of events: cementation, epidermis separation from the cypris cuticle, degeneration of cypris musculature, rotation of the thorax inside the mantle cavity, building of the juvenile musculature, contraction of antennular muscles, raising of the body, shedding of the cypris cuticle, shell plate and basis formation and, possibly, a further moult to become a suspension feeding barnacle. We compare these events with developmental information from other barnacle species and discuss them in the framework of barnacle settlement ecology.

## Introduction

**Figure 1:**
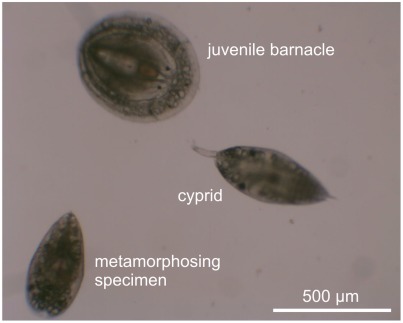
Three developmental stages of the life cycle of *Balanus amphitrite*. An exploring cyprid walking close to a metamorphosing specimen and a juvenile barnacle.

Stalked and acorn barnacles (Cirripedia Thoracica) are important members of marine communities from the rocky intertidal zone to offshore specialized habitats such as coral reefs [1]. A fundamental stage in their complex life cycle is the change from the free-swimming nauplius larva, characteristic of many crustaceans, to life as a permanently attached sessile barnacle. This occurs via a larval stage that is unique to barnacles: the cyprid (Figure 1). The barnacle cyprid is one of the most specialized of marine invertebrate larvae [2], [3], showing little morphological variability across species despite the vast array of adult barnacle body plans.

The settlement stage is clearly of paramount importance in the life cycle of barnacles because a poor choice of surface will often result in death. There has, therefore, been enormous evolutionary pressure on barnacles to make good choices during surface selection and, in many cases, barnacle cyprids are incredibly specific with regard to the substrata onto which they will settle [4]. The settlement stage of the barnacle life cycle is therefore crucial and has been relatively well studied.

Due to the risks implicated in the change of habitat and total remodeling of the body, attachment and metamorphosis of the cyprid stage requires particular specialization [5]. Many acorn (balanomorphan) barnacles, including the model species *Balanus amphitrite* ( = *Amphibalanus amphitrite*) inhabit the intertidal zone (for discussion on the nomenclature of this species, see Clare and Høeg [6] and Carlton and Newman [7]). Facing desiccation during low tide, it is important that the cyprid locates a settlement site and proceeds well into metamorphosis as quickly as possible. Furthermore, it also needs to rapidly commence suspension feeding because, unlike the nauplius, the cyprids is non-feeding and operates with a finite energy reserve. The generalist nature of *B. amphitrite* with regard to its surface preferences and its relatively rapid generation time have also led to it coming into conflict with humans as an important marine fouling organism [8], [9] and these same characteristics have helped to establish the species as a model organism in marine fouling studies.

While the settlement biology of thoracican cyprids and growth and mortality among populations of juvenile barnacles are well studied, surprisingly few papers have dealt with the intervening and very critical series of metamorphic events from cementation to the substratum until the appearance of a suspension feeding juvenile. This is unfortunate, since detailed knowledge on balanomorphan metamorphosis at both morphological and physiological levels could provide explanations both for the high mortality sustained during this period and to possible antifouling measures. Most studies on the morphological changes in balanomorphan metamorphosis either did not use laboratory-reared animals, or else failed to provide precise timing of metamorphic events [10], [11]. Even the widely cited benchmark study of Walley [12] on the profound histological changes seen during metamorphosis of *Semibalanus balanoides* relied on field-sampled specimens without any precise determination of sequence or age. Glenner and Høeg [13], [14] and Takenaka et al. [15] did use laboratory-reared animals but studied only specific details of metamorphosis. For thoracican barnacles, the only in-depth studies providing timing of metamorphic events are those of Kühl [16] and Høeg et al. [17]. In his little cited but surprisingly detailed study, Kühl [16] divided metamorphosis of *Balanus improvisus* into a series of characteristic phases, while Høeg et al. [17] provided video observations on metamorphosis of four cirripede species, including the balanomorphan *Megabalanus rosa*. At the physiological level, studies on the factors that control metamorphic events are almost entirely absent (but see, e.g., [18]), although such information could be critical to understanding barnacle recruitment in general as well as benefiting antifouling studies.

In this paper we present a time line and description of morphological events from settled cyprid to juvenile barnacle in the model species *Balanus amphitrite*, representing an important step towards both a broader understanding of the settlement ecology of this species and a platform for studying the factors that control its metamorphosis. We use digital video microscopy on laboratory-reared cyprids to document the sequence of metamorphosis and confocal laser scanning microscopy (CLSM) to visualize changes in the metamorphosing cyprids while still enclosed within their carapace.

## Materials and Methods

Cyprids of *Balanus amphitrite* were obtained from an established laboratory culture [19] originally collected at Duke University Marine Laboratory, Beaufort, North Carolina (USA). A total of 54 settled cyprids were individually followed at room temperature for usually more than one day and more than 5 hours of videos were recorded. Video recordings were made of cyprids in a Petri dish containing 0.45 µm filtered seawater using an Olympus® CKX-41 inverted microscope equipped with a uEye® digital video camera. In some Petri dishes small rocks or pieces of cover slip were included to promote cyprids to settle in different orientations. Videos were edited using Windows Movie Maker®, ACDSee® ver. 9.0 and Xilisoft Video Converter Ultimate 6®. For CLSM, specimens were fixed in 2.5% glutaraldehyde buffered in filtered seawater, digested in KOH (5% in water, overnight at 50°C), washed in PBS with 0.3% Triton-X 100, cleared with acetic acid and mounted in glycerol. Observations were performed with a Leica DM IRBE microscope equipped with a Leica TCS SP2 confocal laser scanning unit using a 543 nm helium/neon laser and a 570 nm long pass emission filter.

## Results

**Figure 2:**
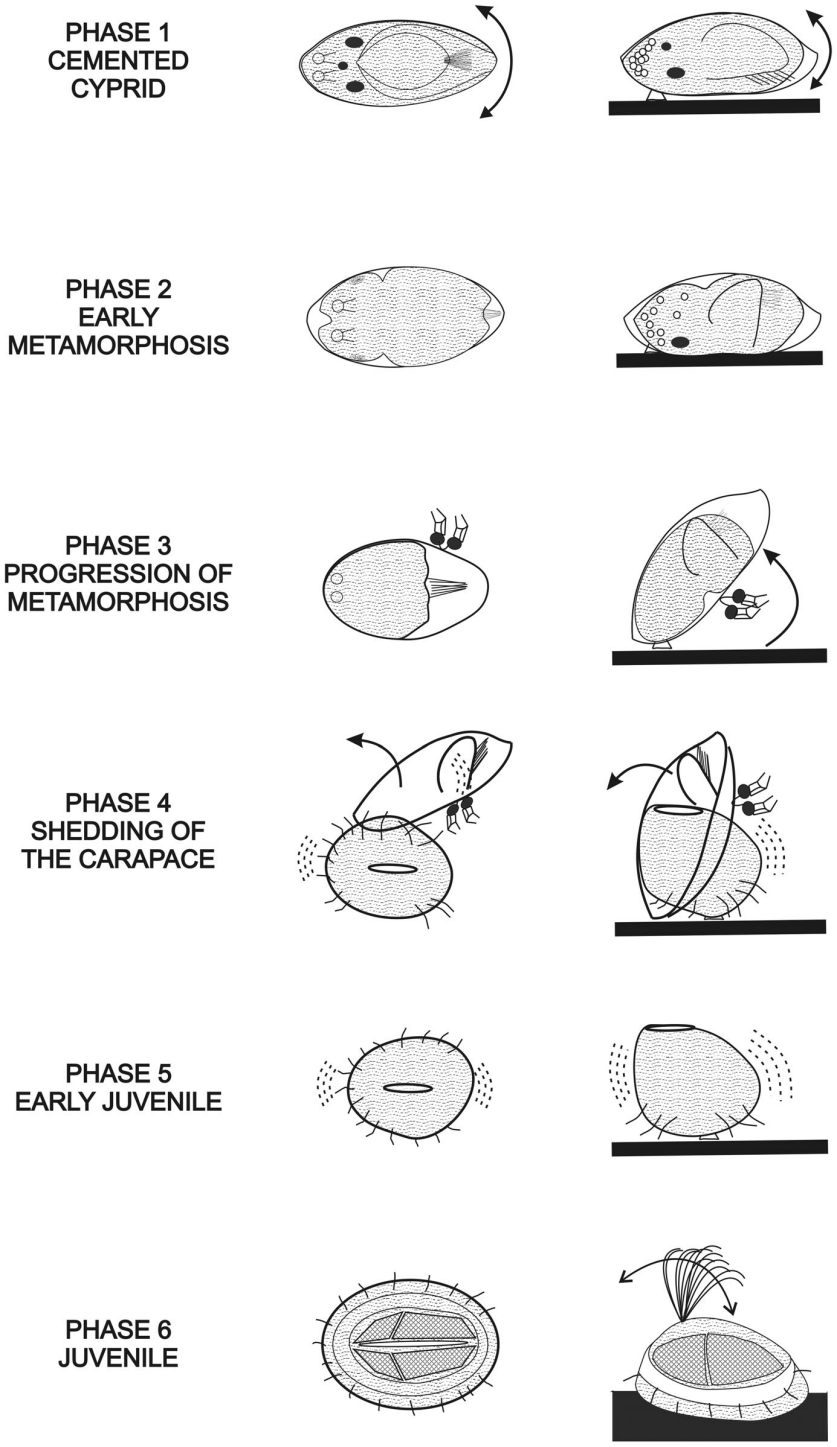
The six phases of metamorphosis of *Balanus amphitrite*. All specimens are schematically drawn from dorsal (central column) and lateral (left column) view. See Table 1 for more details. Phase 1, cemented cyprid: the specimen can still move its body by contractions of the antennular muscles as in exploring cyprids (arrows). Phase 3, progression of metamorphosis: the specimen’s body is raised from the substratum (arrow). Phase 4, shedding of the carapace: carapace slides off the juvenile through the ventral opening of the mantle cavity (arrows). Phase 6, juvenile: long and annulated thoracopods extended well outside the mantle cavity for feeding (arrow).

Kühl [16] divided the metamorphosis of *B. improvisus* into distinct phases based upon his observations using light microscopy. Here we use a modification of his system to similarly divide into six phases the sequence of events in the metamorphosis of *B. amphitrite* from the cemented cyprid to the suspension-feeding juvenile. The duration and main features of the six phases are presented in Figure 2 and Table 1. Clearly, as metamorphosis is a continuous process, this division into phases is intended merely as a useful descriptive tool.

**Table 1:** Duration and main features of the six phases of metamorphosis of *Balanus amphitrite*.

	Duration	Main features
Phase 1– Cemented cyprids	Few hours	Cementation; movements of whole body (as in exploring cyprid); “oil cells” concentrated in anterior body part
Phase 2– Early metamorphosis	About 4 hours	Cypris carapace parallel and tightly applied to the substratum; no more movements of whole body; body contractions inside carapace; separation of epidermis from carapace; rotation of thorax inside carapace; contraction of antennular muscles result in 1) body pulled closer to substratum assuming a lower and broader profile, 2) reduction of anterior mantle cavity and, 3) antennules and compound eyes pressed against the substratum with antennular cuticles irreversibly bent and compressed; “oil cells” dispersed
Phase 3– Progression of metamorphosis	1–4 hours	Raising of body from substratum; pendular and rotation-like movements around attachment point; continued body contractions; antennular cuticles and paired compound eyes expelled, lying outside and only loosely connected to carapace
Phase 4– Shedding of carapace	2–30 minutes	Strong pendular movements and contractions result in shedding of cyprid carapace and thorax
Phase 5– Early juvenile	About 24 hours	Flexible bag-like body shape; thin cuticle allowing body contractions; attachment by cypris cement only, allowing some rotary and pendular movements; short, non-annulated thoracopods, beating but barely extending from mantle aperture; hirsute setae not permanently touching substratum; beginning of shell plate formation
Phase 6– Juvenile	–	Shape of an adult barnacle; whole ventral surface in contact with substratum; long, annulated thoracopods functioning as cirri; shell plates clearly visible

### Phase 1– Cemented Cyprid

**Figure 3:**
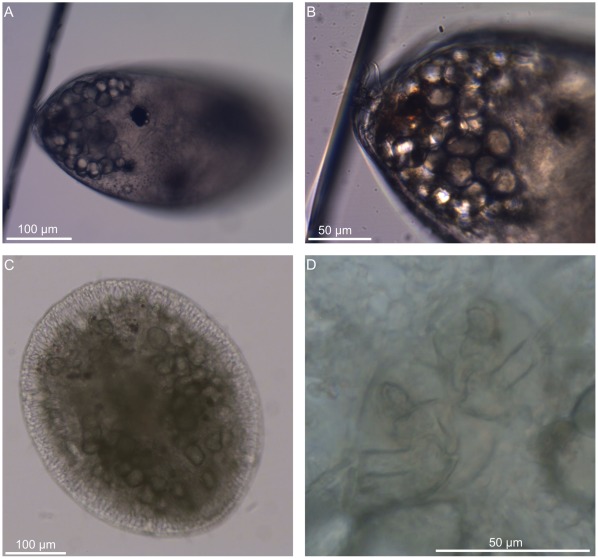
Attachment of a recently cemented cyprid and of an early juvenile. A, a recently cemented cyprid, lateral view. B, higher magnification of A showing the attachment point of the antennules. C, an early juvenile. D, higher magnification of C showing the attachment point of the antennules.

See Figures 2, 3 and Videos S1, S2.

Immediately after irreversible attachment by cement secretion the cyprid shows few, if any, morphological changes compared to the free-swimming form. The cemented cyprid even continues with the same body movements of a still-exploring larva throughout this phase (Videos S1, S2). It is often difficult, therefore, to ascertain whether a stationary larva is still testing the substratum (see [20], [21] for more details) or has permanently cemented itself if the adhesive cannot be observed.

### Phase 2– Early Metamorphosis

**Figure 4:**
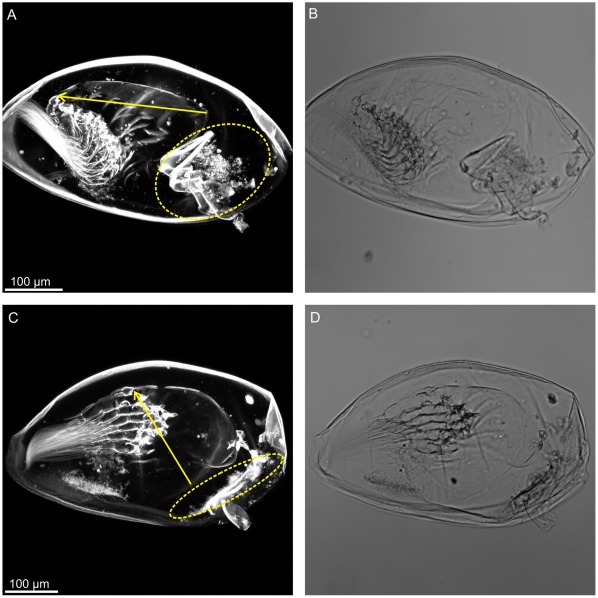
The rotation of the thorax inside the carapace. A, CLSM image of a cemented cyprid showing the thorax with its anterior-posterior axis (arrow) more or less parallel to the anterior-posterior axis of the carapace and a wide anterior mantle cavity (dotted circle) hosting the antennules (very bright in the picture) and the compound eyes (not clearly visible in the picture). B, same as A with bright field. C, CLSM image of a specimen in early metamorphosis (phase 2) showing the rotation of the thorax inside the carapace (arrow identifies approximately the same axis as in A) and a much reduced anterior mantle cavity (dotted circle). D, same as C with bright field.

See Figures 2, 4 and Videos Videos S2, S3.

During this phase (see also [22]) the carapace of the cyprid retains the orientation observed at cementation, viz., parallel and tightly applied to the substratum, but the movements seen during both substrate exploration and Phase 1 now cease (Video S2). After a still period, they are replaced by contractions within the body, never seen in the free larva, which will eventually result in the shedding of the cypris cuticles.

The first morphological change visible by light microscopy is separation of the epidermis from the carapace cuticle anteriorly (Video S2), posteriorly (Videos S2, S3) and to a lesser extent also laterally. This separation signals the incipient moult from cyprid to juvenile and increases in magnitude throughout this phase. Inside the carapace, the thorax now starts to rotate slowly so that the original ventral surface carrying the thoracopods assumes a posterior orientation (Figure 4).

Simultaneous to the rotation of the thorax, the antennular muscles start to contract. In a free-swimming cyprid this would result in retraction of these appendages into the anterior mantle cavity but once permanently fixed to the surface, the muscular contraction instead results in several irreversible changes. The contraction pulls the whole cypris body even closer towards the substratum. This results in splaying of the two sides of the flexible carapace, whence the metamorphosing body assumes a lower and broader profile than in the free larva (Video S2). Further, the spacious anterior mantle cavity, normally housing the antennules (Figure 4a, b) and the compound eyes [12], is compressed towards the surface and is thereby completely obliterated (Figure 4c, d). The effect of this is that the antennules and compound eyes are now pushed tightly against the surface beneath the body. Finally, the complex antennular cuticles are irreversibly dislocated. During this phase the anteriorly situated, large and globular ‘oil cells’ become dispersed and therefore difficult to trace in live cyprids (see also [12]).

### Phase 3– Progression of Metamorphosis

See Figure 2 and Videos S3, S4.

The onset of this phase is signaled by the raising of the cyprid so that it now subtends an acute angle with the substratum (Video S2). This is accompanied by pendular and rotational movements around the attachment point (Videos S3, S4). The contractions, already present in phase 2, become frequent. At this stage the muscles of the cyprid have largely degenerated [12], [14]. Therefore these movements, never seen in a non-metamorphosing cyprid, are probably caused by the emerging muscles of the juvenile barnacle. During phase 3, the antennular cuticles and the paired compound eyes are both expelled together as one structure, coming to lie outside but still loosely connected to the carapace. Linked together, these expelled cuticles very much resemble a ‘pair of old spectacles’. Toward the end of phase 3, the above mentioned circular and pendular movements become stronger and almost continuous (Videos S3, S4).

### Phase 4– Shedding of the Carapace

See Figures 2, 3 and Videos S3, S4, S5.

Due to the body movements of the metamorphosing specimen, the spent cypris carapace now begins to slide off the juvenile through the ventral opening of the mantle cavity. The strong body movements eventually result in the complete elimination of the carapace together with the loosely attached thorax, eyes and antennular cuticles.

At this stage the slit-like mantle aperture of the emerging juvenile is bordered on both sides by narrow crests lined with a row of conspicuous setae (Videos S3, S4; see [13] for details). After shedding the carapace, these crests remain present in the early juvenile until it assumes the shape of an adult barnacle.

### Phase 5– Early Juvenile

See Figures 2, 3 and Videos S4-S6.

Immediately after shedding the carapace, the juvenile has a very thin cuticle. It resembles an upright and very flexible bag still attached only by the tiny blob of original cypris cement, around 100 µm in diameter depending on surface, embedding the cypris attachment discs (Figure 3, Videos S4, S5). The thin cuticle allows the whole body to continue with the contractile and pendular movements initiated during the previous phase (Videos S4, S5). Accordingly, the early juvenile has little resemblance to an adult barnacle except for the beating of the thoracopods and the presence of an apical mantle aperture through which the tips of these appendages may occasionally be seen (Video S5).

The cuticle of the cypris thoracopods is shed together with the carapace, but the thoracopods of the early juvenile do not yet have the annulated shape of the later so-called cirri, used for suspension feeding [23]. Instead, they seem to consist of a small number of segments as also found in the cyprid. These short thoracopods beat more or less continuously, but are not yet extended from the mantle cavity (Videos S4, S5) and the main function is probably to produce a current for respiration. Barnacles are, however, known to perform microfiltration even when the cirri are retained in a closed mantle cavity, so we cannot preclude that even the early juvenile may have started feeding in this way on very small organisms.

The hirsute (peripheral) setae (sometimes called “hirsute hairs”, e.g., [24]) characteristic of juvenile barnacles are visible at this stage, but none of them permanently touch the substratum (Video S4) (see also [13]).

During this phase the barnacle body gradually begins to assume the shape of a flat cone with an apical mantle aperture, the whole ventral surface starts to be in contact with the substratum and shell plate formation begins. The first sign of shell plate formation is the appearance of two clefts demarcating the position of the rostrum at one end of the juvenile (Video S4).

### Phase 6– Juvenile

See Figures 2 and Video S6.

During the following 24 hours the juvenile assumes the shape of an adult barnacle and the whole ventral surface (about 400 µm in diameter) is in contact with the substratum. The thoracopods are now long and annulated and the three posterior pairs can be extended for feeding as a true cirral basket (Video S6). The very different morphology and annulation of the thoracopods suggests a moult between phase 5 and 6. The shell plates are now better developed but their formation falls beyond the scope of our study (see [22], [25]–[27]).

## Discussion

Our observations show that metamorphosing specimens of *B. amphitrite* reach the juvenile stage about 32 hours after irreversible attachment. During this period the metamorphosing barnacle must be very susceptible to environmental effects such as water currents (e.g., [28]) and desiccation (e.g., [29]). As juveniles (our phase 6), they are more firmly attached and increasingly protected by the developing shell plates, but in *B. amphitrite* it seems that this stage cannot be reached within a single high tide period. It would therefore be interesting to compare the course and speed of metamorphosis in balanomorphan barnacles living at different zones in the tidal range and establish if there is any correlation between exposure and speed of metamorphosis. Individuals of *Megabalanus rosa*, a balanomorphan that inhabits the upper tidal zone, reach the early juvenile phase in about the same time as *B. amphitrite* but in species of the pedunculated barnacle *Lepas*, which are submerged throughout metamorphosis, the whole process lasts several days [17]. These observations suggest that environmental pressures in the intertidal zone may have substantially influenced the speed of metamorphosis.

There are, of course, many factors other than desiccation and energy exhaustion that may influence the speed of metamorphosis in barnacles. Of the ∼1000 extant species of described barnacles, ∼250 are parasitic (mainly on decapods) and this lifestyle is therefore well represented in the group. Parasitic barnacles face different, but equally serious pressures during their metamorphosis from cyprid to juvenile. In parasitic barnacles (Rhizochephala), the whole process of locating a settlement site (host), attaching and metamorphosing into the first juvenile stage can be completed within less than 20 minutes [30]. For cyprids trying to infest a host, the driving factor for this rapid metamorphosis is primarily the risk of removal by the host through grooming. There is a considerable evolutionary advantage for the host to achieve this because if successful, the rhizocephalan will sterilize it [31], [32]. Later, other cyprids may settle on juvenile parasites and here the equally rapid speed of metamorphosis is due to male-male competition [32], [33]. Balanomorphan barnacles, such as *B. amphitrite*, probably face similar challenges in their own ecological niche. The species rich and ecologically important group of coral barnacles (Pyrgomatidae) must, for example, settle and metamorphose through the cnidoblast-armed epidermis of the cnidarian host [34].

In balanomorphans, such as *B. amphitrite*, the most critical phase is probably the early juvenile (phase 5), which is still only loosely attached to the substratum and protected neither by a cypris carapace nor by shell plates. This calls for a study, under field and laboratory conditions, of the mortality during the different metamorphic phases as defined in this paper. Bernard and Lane [35], who also studied metamorphosis in *B. amphitrite,* drew very puzzling conclusions about the early juvenile barnacle that even gained entry to general invertebrate textbooks (e.g., [36]). In their interpretation, this phase was not clad in any cuticle and the hirsute setae surrounding the basis of the juvenile were described as cilia. Glenner and Høeg [13], through use of SEM, already commented on these erroneous conclusions and the present observations clearly demonstrate that the metamorphosing barnacle is at all times entirely covered with cuticle; however, this cuticle is indeed very thin and allows contractile and pendular movements.

From phase 6 (about 24 hours after the shedding of the cypris cuticle), the juvenile is well equipped with shell plates and multi-annulated feeding cirri. The morphology of the cirri at this phase clearly suggests a moult between phase 5 and 6 as an increase in appendage article number without a moult is not known to occur in any arthropod. In adult barnacles only the lining of the mantle cavity and of the body enclosed in the mantle cavity (including appendages) are shed during moulting [1], [37] and, as this cuticle is very thin in the early juvenile, it may well have been missed as a floating exuvium. The profound switch between moulting the whole body (as in nauplius and cyprid) to being armed with mineralized shell plates and moulting only the mantle cavity is something that has never been investigated at the physiological level. Presumably, this shift also involves fundamental physiological changes and would be an interesting research target.

Despite the obvious importance of the cyprid to juvenile metamorphosis in elucidating the evolution of barnacles and understanding, and eventually controlling, their colonization of both natural and man-made surfaces, the physiological mechanisms regulating this process are currently unclear. In arthropods, moulting and metamorphosis are regulated by two classes of hormones: ecdysteroids and sesquiterpenoids. There are several ‘traditional’ studies on barnacle endocrinology (reviewed in [38]) but our knowledge here is far from comparable to what is known about insects and malacostracan crustaceans. Both ecdysone and methyl farnesoate (the major sesquiterpenoid crustacean hormone, more or less corresponding to the ‘more famous’ insect juvenile hormone) have been isolated in *B. amphitrite* and their role in the regulation of metamorphosis is certain, although unclear because their effects seem to be concentration-dependent [18], [39], [40]. Studies on barnacle physiological regulation rely also on identifying and comparing particular stages. So far, only obviously different stages have been employed. Thiyagarajan and Qian [41] compared the overall profile of protein expression between nauplius, swimming cyprid, metamorphosing cyprid and juvenile, but extending such studies to the different phases of the metamorphic process may also be highly informative. *B. amphitrite*, the most studied barnacle species at the molecular level (an extensive EST database and some reference genes for qRT-PCR are available [42]–[44]), would be the obvious choice and our timeline of metamorphic events in this species now provides a platform for such studies.

Although pedunculated and acorn barnacles (Thoracica) are celebrated models for invertebrate metamorphosis, there is still a surprising scarcity of detailed studies of this process. Physiological studies of metamorphosis are almost entirely absent and at the morphological level no detailed account exists for any of the numerous and biologically diverse pedunculated species. Thoracican barnacles inhabit a wide range of substrata including forms that penetrate into the living tissues of corals and whales [1], [34]. This diversity suggests that metamorphosis may vary considerably more than presently believed and be optimized for specific niche requirements. In species of the pedunculated barnacle *Lepas*, the scant available information indicates that metamorphosis differs in several important respects from that seen in balanomorphans such as *B. amphitrite*. These differences concern both the orientation of the body during the process and the method by which the carapace is eliminated [17]. Aside from Walley [12], the only other complete accounts of morphological changes during cypris metamorphosis concern the parasitic barnacles (Rhizocephala), where the process results in the injection of a highly reduced endoparasitic stage into the blood system of the host, typically a decapod crustacean [17], [30], [45], [46].

Metamorphosis in *B. amphitrite* involves a complex sequence of events. Our video observations clearly showed cementation and uneven epidermis separation from cuticle, degeneration of cypris muscles (as suggested by the end of cyprids-like movements from phase 2), rotation of the thorax inside the posterior mantle cavity, building of the juvenile muscles (as suggested by the body contractions from phase 4), contraction of antennular muscles, raising of the body and finally shedding of the cypris cuticle. The early juvenile then undergoes further changes to become a feeding barnacle enclosed in shell plates. Video observations cannot provide more than this (but see [12] for more histological changes on another balanomorphan species), nevertheless, this description provides some easy to observe morphological details to assess in which phase of the metamorphic process a given specimen is. Along with detailed microscopic studies, future research on cirripede metamorphosis should also include methods from physiology and developmental biology to understand the underlying biochemical control processes and the extent to which these may have diverged within the group. Only based on such comprehensive information can we hope to understand the evolution of metamorphosis within the highly successful group of cirripedes.

## Supporting Information

Video S1 **Early phase 1.** Recently cemented cyprid in a Petri dish filled with small stones. The cyprid’s thoracopods are still actively beating and the antennular movements (contractions of antennular muscles) result in movements of the whole cypris body.
(WMV)

Video S2 **Phase 1 and phase 2 in dorsal view.** This sequence shows how a recently cemented specimen, still moving its body as an exploring cyprid, gradually becomes more stationary and begins to exhibit separation between cuticle and tissue anteriorly in the body. It is also becomes applied closely to the substratum, thus assuming a broader body outline.
(WMV)

Video S3 **Phases 2–5, lateral view.** The sequence follows a specimen from the end of phase 2 (body starts to be raised from the substratum) to phase 5 (early juvenile). Note the movements and contractions of the body that result in the shedding of the cypris carapace.
(WMV)

Video S4 **Phases 3–5, dorsal view.** The sequence shows a specimen from when it is already raised (phase 3) to the early juvenile (phase 5).
(WMV)

Video S5 **Phase 4 and phase 5, lateral view.** The sequence shows a lateral view of the shedding of the cypris carapace and the beating of the very short thoracopods (“cirri”) of the early juvenile.
(WMV)

Video S6 **Phase 5 and phase 6, dorsal view.** The sequence shows the marked difference in thoracopod (“cirri”) length and annulation from the early juvenile (phase 5) to the juvenile (phase 6).
(WMV)
